# Automatic segmentation of optical coherence tomography pullbacks of coronary arteries treated with bioresorbable vascular scaffolds: Application to hemodynamics modeling

**DOI:** 10.1371/journal.pone.0213603

**Published:** 2019-03-14

**Authors:** Marco Bologna, Susanna Migliori, Eros Montin, Rajiv Rampat, Gabriele Dubini, Francesco Migliavacca, Luca Mainardi, Claudio Chiastra

**Affiliations:** 1 Laboratory of Biological Structure Mechanics (LaBS), Department of Chemistry, Materials and Chemical Engineering “Giulio Natta”, Politecnico di Milano, Milan, Italy; 2 Department of Electronics, Information and Bioengineering, Politecnico di Milano, Milan, Italy; 3 Center for Advanced Imaging Innovation and Research (CAI2R), and the Bernard and Irene Schwartz Center for Biomedical Imaging, Department of Radiology, New York University School of Medicine, New York, New York, United States of America; 4 Sussex Cardiac Centre, Brighton and Sussex University Hospitals, Brighton, United Kingdom; 5 PoliTo^BIO^Med Lab, Department of Mechanical and Aerospace Engineering, Politecnico di Torino, Turin, Italy; San Giovanni Addolorata Hospital, ITALY

## Abstract

**Background / Objectives:**

Automatic algorithms for stent struts segmentation in optical coherence tomography (OCT) images of coronary arteries have been developed over the years, particularly with application on metallic stents. The aim of this study is three-fold: (1) to develop and to validate a segmentation algorithm for the detection of both lumen contours and polymeric bioresorbable scaffold struts from 8-bit OCT images, (2) to develop a method for automatic OCT pullback quality assessment, and (3) to demonstrate the applicability of the segmentation algorithm for the creation of patient-specific stented coronary artery for local hemodynamics analysis.

**Methods:**

The proposed OCT segmentation algorithm comprises four steps: (1) image pre-processing, (2) lumen segmentation, (3) stent struts segmentation, (4) strut-based lumen correction. This segmentation process is then followed by an automatic OCT pullback image quality assessment. This method classifies the OCT pullback image quality as ‘good’ or ‘poor’ based on the number of regions detected by the stent segmentation. The segmentation algorithm was validated against manual segmentation of 1150 images obtained from 23 *in vivo* OCT pullbacks.

**Results:**

When considering the entire set of OCT pullbacks, lumen segmentation showed results comparable with manual segmentation and with previous studies (sensitivity ~97%, specificity ~99%), while stent segmentation showed poorer results compared to manual segmentation (sensitivity ~63%, precision ~83%). The OCT pullback quality assessment algorithm classified 7 pullbacks as ‘poor’ quality cases. When considering only the ‘good’ classified cases, the performance indexes of the scaffold segmentation were higher (sensitivity >76%, precision >86%).

**Conclusions:**

This study proposes a segmentation algorithm for the detection of lumen contours and stent struts in low quality OCT images of patients treated with polymeric bioresorbable scaffolds. The segmentation results were successfully used for the reconstruction of one coronary artery model that included a bioresorbable scaffold geometry for computational fluid dynamics analysis.

## 1. Introduction

Coronary artery disease is the leading cause of death worldwide [1]. Balloon angioplasty followed by stent implantation is the main treatment strategy of diseased coronary arteries [1]. Although in the short-term such treatment is generally effective, in the medium- and long-term stent efficacy can be reduced by adverse clinical events, such as in-stent restenosis and thrombosis [2]. Multiple causes have been associated with those events. Among them, experimental and *in vivo* evidences suggest that the altered local fluid dynamics induced by the stent plays an important role on promoting in-stent restenosis and thrombosis [3,4].

Computational fluid dynamics (CFD) is a valuable tool for the analysis of the local fluid dynamics in complex three-dimensional (3D) geometries such as the coronary arteries [5]. Clinical imaging can be used for the creation of realistic 3D patient-specific vessel geometries for CFD analysis [5]. In particular, the fusion of intravascular optical coherence tomography (OCT), which allows the visualization of the lumen borders and stent struts at high resolution (axial resolution of 12–15 μm and lateral resolution of 20–40 μm [6]), with X-ray angiography projections, which provide a reference in the 3D space, enables the reconstruction of patient-specific models of coronary arteries including the stent geometry [7].

Manual segmentation of lumen contours and stent struts in OCT images for subsequent 3D modeling is a time-consuming procedure. Therefore, automatic segmentation algorithms are widely employed to detect both lumen and stent [7]. Over the years, particular effort has been applied on the development of algorithms for the detection of metallic drug eluting coronary stents [7]. Nevertheless, these algorithms are not effective for polymeric bioresorbable scaffolds due to the different optical properties compared to the conventional metallic stents [7]. Few studies have proposed a segmentation strategy for polymeric bioresorbable scaffolds so far [8–10]. The limited research activity on the detection of those type of stents in OCT images may be addressed to the recent introduction of this technology into the market and the negative long-term clinical outcomes of the Absorb Bioresorbable Vascular Scaffold (BVS) (Abbott Vascular, Abbott Park, IL, USA) [11]. Nevertheless, the extensive development of the bioresorbable stent technology suggests that the detection algorithm for bioresorbable scaffolds will probably become more and more important in the immediate future [12,13].

The currently available segmentation algorithms of polymeric bioresorbable scaffolds are characterized by good sensitivity and precision [8–10]. Their validation was performed on high quality 16-bit images obtained with the C7-XR OCT system (St. Jude Medical, St. Paul, MN, USA) [8–10] and typically on less than 10 pullbacks [8,10]. Little or nothing is known about the performance of automatic segmentation algorithms on 8-bit images acquired with different scanners. Furthermore, the quality of the OCT acquisition, which can be affected by noise inside the vessel lumen and poor stent strut definition, may influence the performance of the segmentation algorithm. In the present study, a new segmentation algorithm for the detection of both lumen contours and polymeric scaffold struts from *in vivo* 8-bit OCT images is proposed. The algorithm was tested on 23 OCT pullbacks acquired with the Lunawave OCT imaging system (Terumo Corp., Tokyo, Japan). Furthermore, a method for automatic OCT pullback quality assessment is developed. Last, to demonstrate the applicability of the segmentation algorithm for the creation of patient-specific coronary artery models that include a bioresorbable scaffold, one representative case was selected for 3D reconstruction and subsequent CFD analysis after lumen contours and stent struts segmentation.

## 2. Material and methods

### 2.1 Image data collection

Twenty-three *in vivo* OCT pullbacks of coronary arteries treated at the Brighton and Sussex University Hospitals (Brighton, UK) with the Absorb BVS were selected to develop and validate the segmentation algorithm. The OCT scans were performed after stent implantation in patients belonging to the Absorb in Bifurcation Coronary trial (ABC-ONE), a single center, randomized trial comparing different sizing strategies when using bioresorbable scaffolds for the treatment of coronary bifurcation diseases [14]. The study complies with the Declaration of Helsinki on human research and was approved by the South East Coast Research Ethics Committee (Brighton and Sussex, UK). All patients gave written informed consent.

OCT imaging was performed using the FastView catheter (Terumo Corp.) and the Lunawave OCT coronary imaging console (Terumo Corp.). The OCT catheter was carefully advanced over a guidewire beyond the target region. While contrast agent was continuously injected at a rate of 4 ml/s, OCT images of the main coronary vessel were acquired at a rate of 160 frame/s with a pullback speed of 20 mm/s. The contrast agent used was Omnipaque 350 mg/ml (GE Healthcare, Chicago, IL, USA). The in-plane OCT resolution was 15 μm. The longitudinal resolution (i.e. the distance between two consecutive frames) was 125 μm. The OCT frames were saved as 8-bit grey-scale images.

In addition to OCT, multiple angiographic projections were acquired during the stenting procedure.

### 2.2 Lumen and stent segmentation algorithm

**Fig 1:**
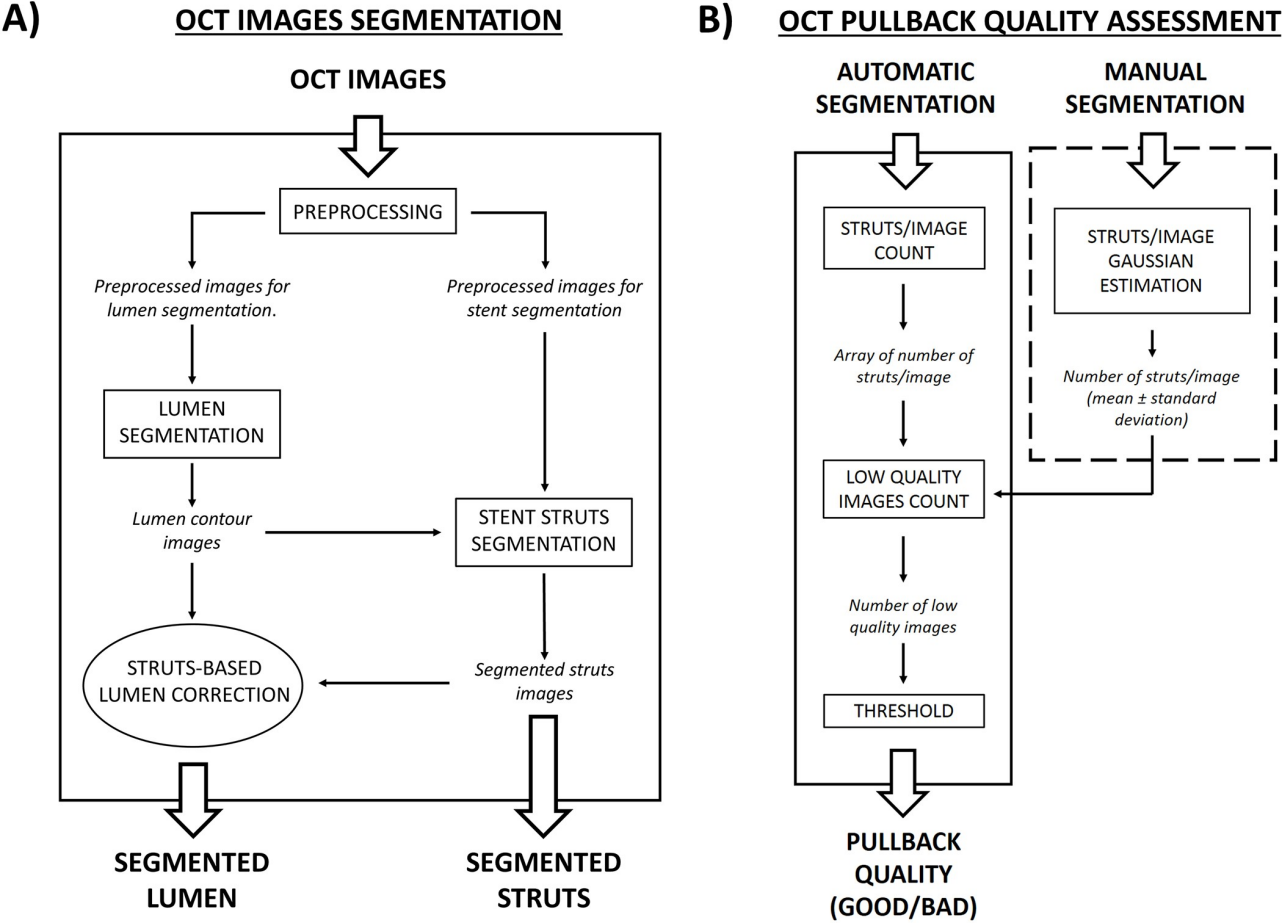
Flow-charts of the algorithms presented in this study. A) Main steps of the segmentation algorithm. The operations presented in square blocks are always performed, while that in the elliptic block is optional. B) Main steps of the OCT pullback quality assessment. The operation reported in the dashed box are performed only once on the manual segmentation, while the others are performed for every pullback. The output of each step is always written in italic.

The workflow of the OCT image automatic segmentation algorithm, which enables the detection of both lumen contours and polymeric stent struts, is shown in Fig 1A. The algorithm comprises four main steps, namely (1) image pre-processing, (2) lumen segmentation, (3) stent segmentation, and (4) struts-based lumen correction. Image segmentation was performed in MATLAB 2016b (Mathworks, Natick, MA, USA) on a desktop computer equipped with CPU i7-950 @3.07 GHz and 16 GB of RAM.

#### 2.2.1 Image preprocessing

**Fig 2:**
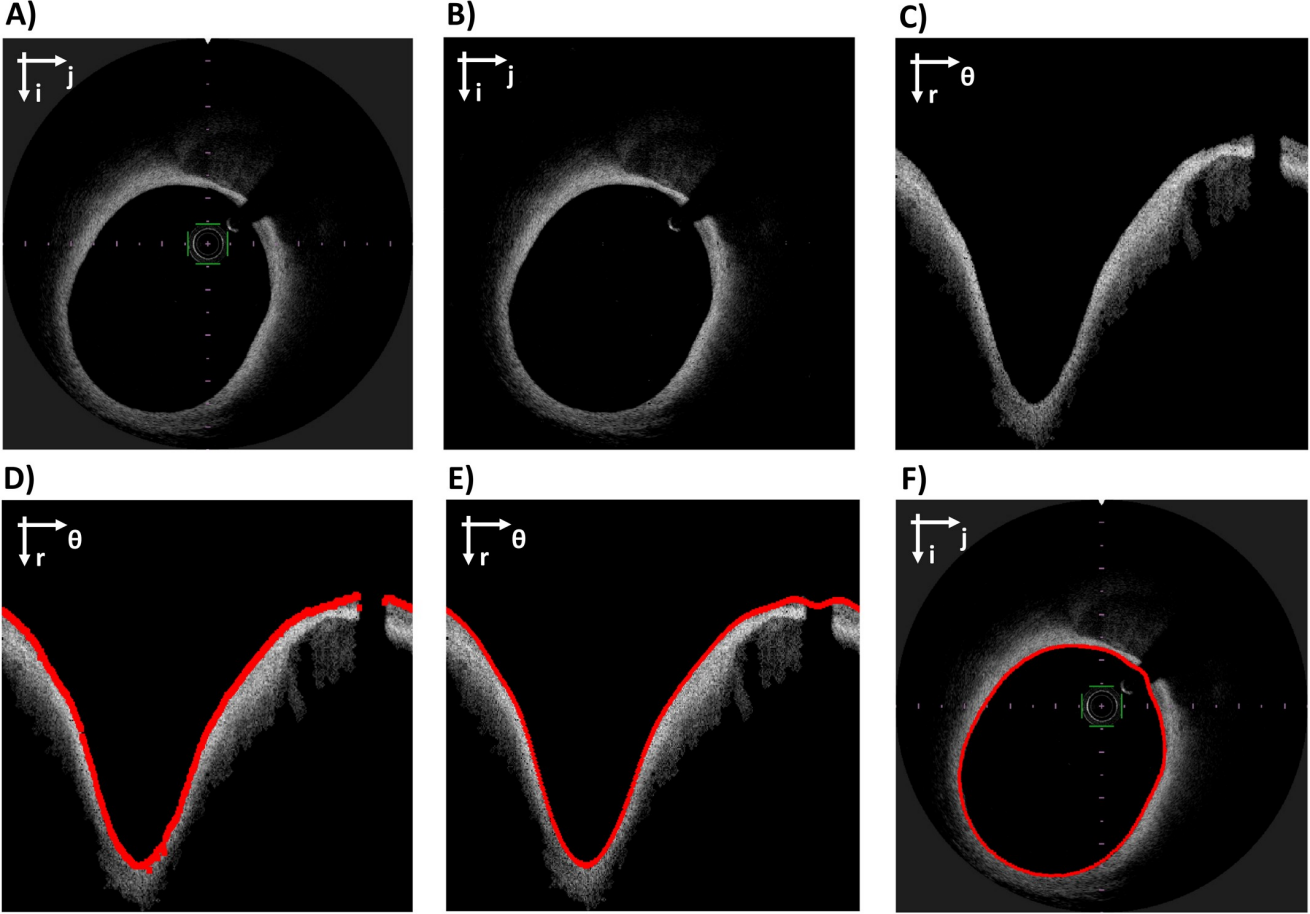
Workflow of lumen segmentation. A) Original OCT image in Cartesian coordinates. B) Image after pre-processing. C) Image in polar coordinates. D) Raw lumen contour detection (red). E) Lumen contour after interpolation (red). F) Original image in Cartesian coordinates with the segmented lumen contour (red). The Cartesian coordinate system (i; j) or the polar coordinate system (r; θ) is indicated on the top left of each image.

**Fig 3:**
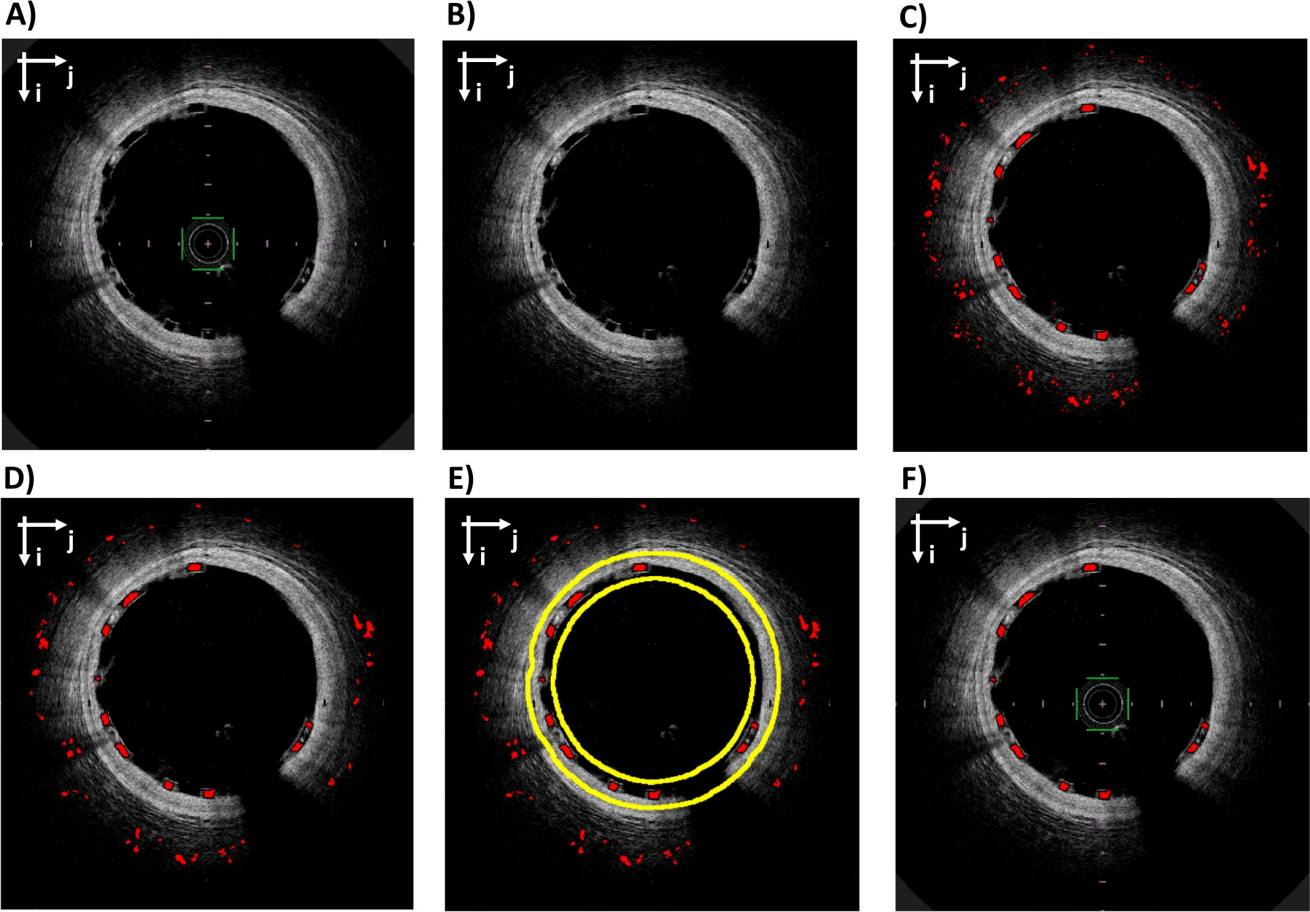
Workflow of stent strut segmentation. A) Original OCT image in Cartesian coordinates. B) Image after pre-processing. C) Rough stent struts segmentation (red). D) Segmented struts after area constrain. E) Segmented struts (red) after area constrain with superimposed the boundaries of the region of maximum likelihood for stent struts (yellow). F) Original image with final stent struts segmentation (red). The Cartesian coordinate system (i;j) is indicated on the top left of each image.

The pre-processing step is similar to the one described in [15]. Briefly, the calibration bar of the visualization system and the OCT catheter are removed. A further processing step, which is necessary only before lumen segmentation, is the removal of the noise inside the lumen. The image without noise is obtained by product of the original image and a binary mask. The binary mask is created through the following steps: (1) intensity thresholding (the quantile 0.85 of the pixel intensity distribution is used), (2) morphological opening (MATLAB function *imopen*), (3) area thresholding (MATLAB function *bwareaopen*). Examples of pre-processing are shown in Figs 2A, 2B, 3A and 3B.

#### 2.2.2 Lumen segmentation

The lumen segmentation step is the same of [15] (Fig 2). Briefly, the pre-processed images are converted into polar coordinates (r, θ) and a Sobel filter is applied to detect edge pixels. The first edge pixel for each A-scan (i.e. one-dimensional depth profile) is considered as part of the lumen contour. The identified pixels are interpolated, and the obtained curve is converted back to Cartesian coordinates.

#### 2.2.3 Stent segmentation

The workflow of stent segmentation is shown in Fig 3. The Absorb BVS struts appear in the OCT images as approximately rectangular regions with a low intensity core surrounded by bright pixels. Ideally, such struts can be identified by following three steps: (1) intensity thresholding (the quantile 0.85 of the pixel intensity distribution is used); (2) flood fill operation to close the holes in the binary image (performed with MATLAB command *imfill*); (3) Boolean subtraction of the original binary image from the filled image. However, when the stent struts have only a partially connected layer of bright pixels at their boundary (i.e. “open box” struts), the procedure described above is not able to recognize the struts. To reduce the problem introduced by the presence of “open box” struts two operations are performed:

- application of a nonlinear filter (γ-filter, with γ = 0.6) before “step (1)” to enhance the contrast of low grey-level.
- morphological closing before “step (2)”, with a disk structure element of 1-pixel radius.

These operations increase the sensitivity of the segmentation. However, they generally fail to large gaps in the struts boundary and may increase the number of false positives, especially close to the outer wall.

The false positives are removed by applying a series of filtering rules (Fig 3D–3F). Firstly, the struts are bounded to have area between two thresholds *A*_*min*_ and *A*_*max*_ (10 and 300 pixels, respectively). Secondly, among the remaining struts, only those inside a region of maximum likelihood are kept. The region of maximum likelihood is defined as the intersection between two regions. The first region is obtained by applying dilatation to the previously detected lumen contours (disk structure element with radius of 20 pixels). The second region is obtained by finding a reference line and by performing its dilatation (disk structure element with radius of 5 pixels). The reference line is a spline that interpolates the centroids of the struts identified by a first rough segmentation. This segmentation is performed by following the steps 1–3, as previously described, followed by a morphological opening with a 3-pixels radius disk. This allows the execution of a low-sensitivity but high-specificity segmentation, which is ideal for the interpolation of the reference line. This line is interpolated by using also the struts identified in the images adjacent to the one of interest (similarly to [15]), to account for images with a low number of struts, where the quality of the interpolation is low.

#### 2.2.4 Struts-based lumen correction

**Fig 4:**
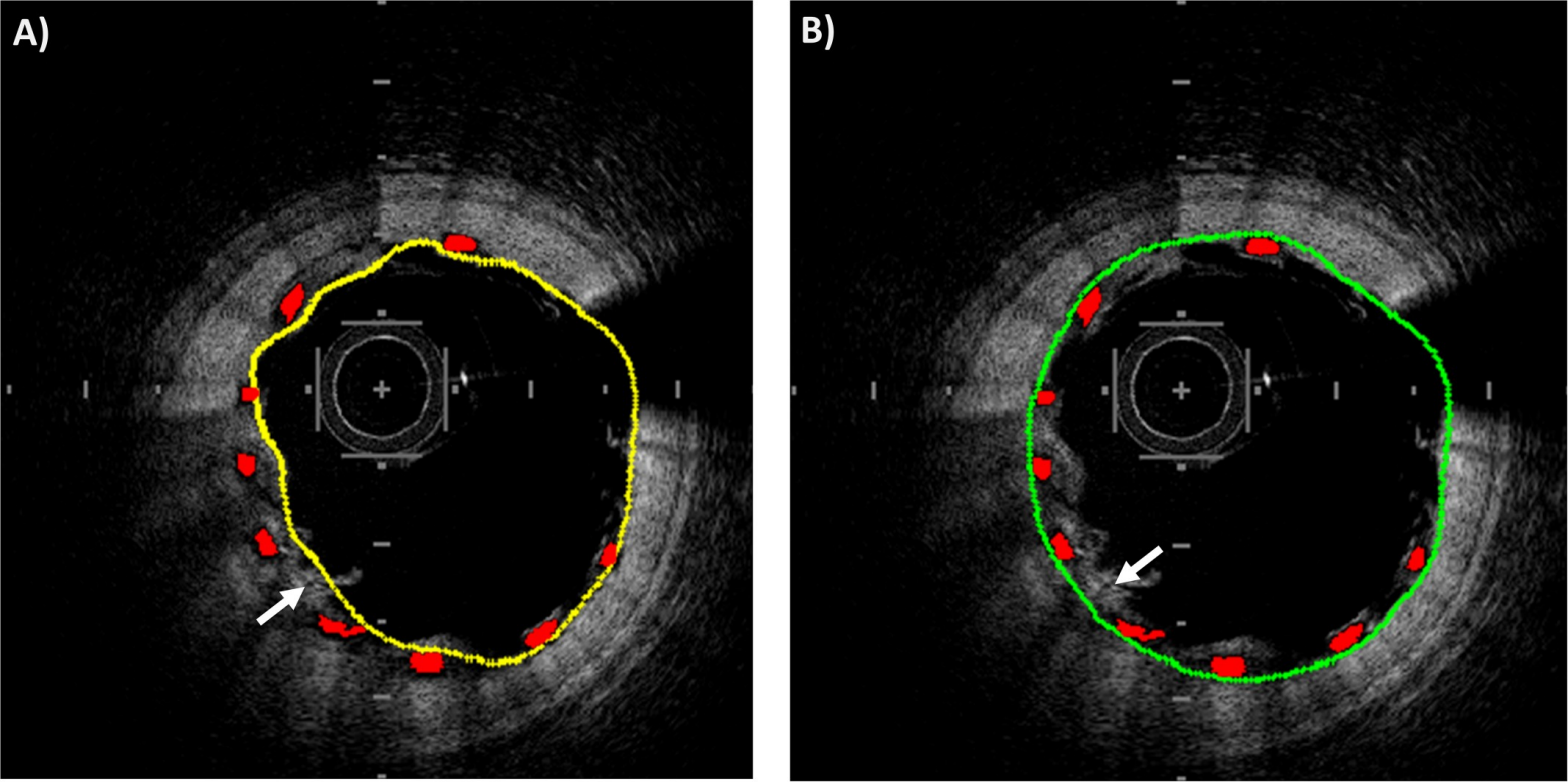
Example of struts-based lumen correction. A) Lumen contour before correction (yellow) and stent struts (red). B) Lumen contour after the correction (green) and stent struts (red). In both the images, a white arrow points to an example of blood residual close to stent struts that may alter the quality of lumen segmentation.

In case of baseline acquisitions (i.e. immediately post-stenting acquisition) and well-apposed stent struts, the adluminal wall of each strut is expected to lay on the lumen boundary. However, when two struts are in contact or residual blood is present next to the lumen (Fig 4A), the contour detection is not accurate because the first bright pixels are wrongly associated to the lumen boundary. The stent struts are used as a reference to correct the lumen contour. First, the binary images with lumen contour and identified struts are converted to polar coordinates. Secondly, the image with the stent strut is dilated using a rectangular structure element (size 1x10 pixels in radial and tangential directions, respectively). Thirdly, the lumen contour image and the stent strut image are superimposed and, for each A-scan, the white pixel with the highest radial coordinate is selected. Lastly, the identified points are corrected to obtain the final lumen contour (Fig 4B).

Unlike all the other phases of lumen segmentation, the struts-based lumen correction is optional. While this correction is mandatory when baseline OCT images are analyzed, in case of follow-up OCT images, where the stent struts are covered by the neointimal, the segmentation algorithm can either be used without correction to detect the follow-up lumen or with correction to identify the baseline lumen contour [16].

### 2.3 Algorithm for OCT pullback quality assessment

**Fig 5:**
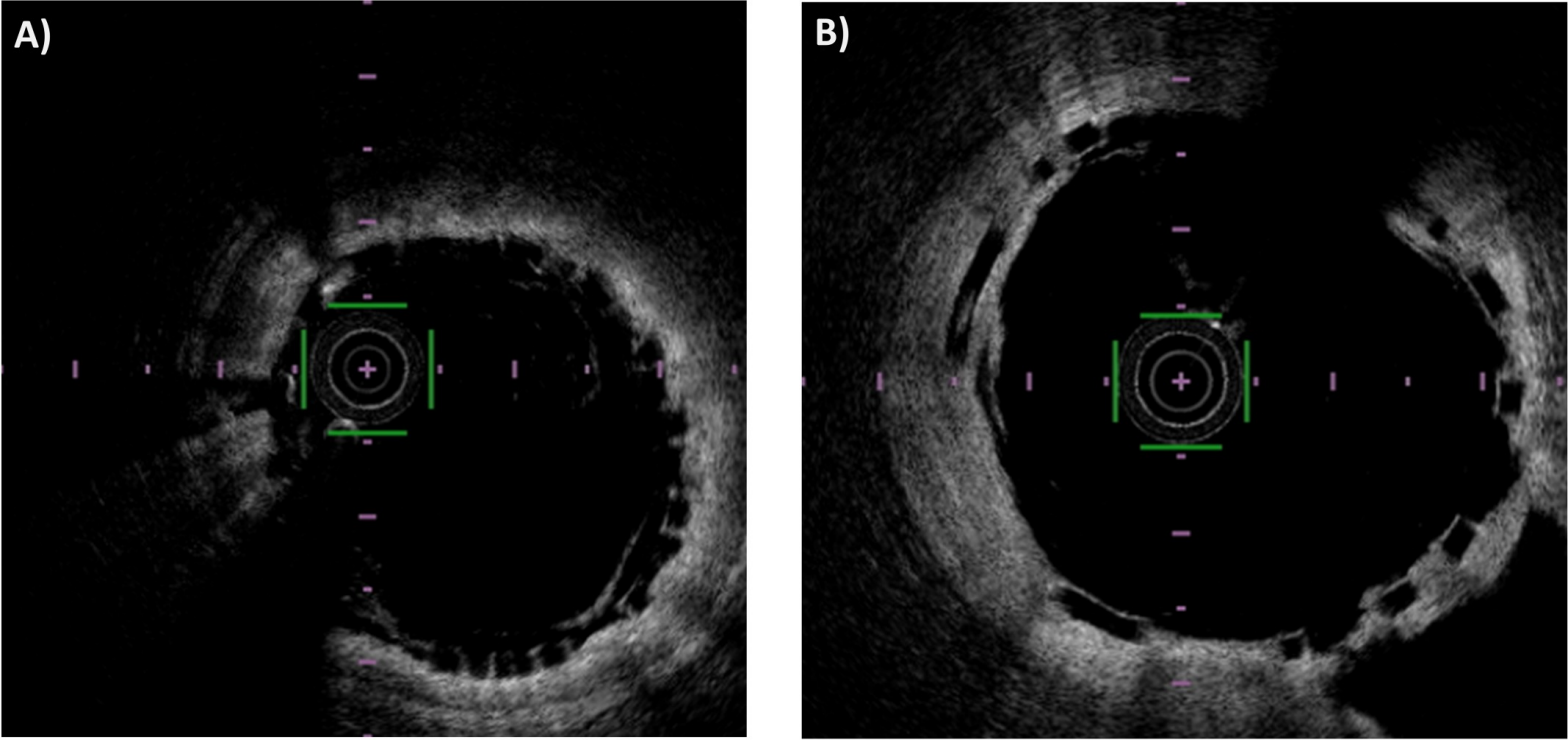
Example of ‘poor’ and ‘good’ quality image. A) In the ‘poor’ quality image, the struts are recognizable by human eye but do not have the preserved box shape. B) In the ‘good’ quality image, the struts are recognizable and have a clearly defined box shape.

The OCT pullbacks acquired for this study are characterized by different image quality in terms of definition of the stent struts. If the stent strut ‘box shape’ is almost absent or there are too many blood residuals inside the lumen, the image quality is classified as ‘poor’ (Fig 5A). If stent strut ‘box shape’ is recognizable, the image quality is classified as ‘good’ (Fig 5B) and the stent strut segmentation algorithm is expected to work properly.

Here, we propose an automatic method able to evaluate the OCT pullback image quality at the end of the segmentation process. This method classifies the OCT pullback image quality as ‘good’ (good segmentation results are expected) or ‘poor’ (therefore, poor segmentation results are expected). It comprises the steps shown in Fig 1B and is described below.

The first step of the algorithm is the estimation of the number of expected struts per image. This number depends on the stent design and is expected to follow a Gaussian distribution across frames. By analyzing a manual segmentation of one or more OCT pullbacks containing a stent, it is possible to estimate the Gaussian distribution of the expected strut number in each image and its parameters (i.e. the mean and standard deviation). In this study, we estimated the parameters of the Gaussian related to the Absorb BVS by using the manual segmentation performed for the validation of the segmentation method (details are reported in subsection 2.4).

Once the automatic segmentation has been performed, the number of regions detected in each image can be counted. If the number of detected regions is below the 5% quantile of the Gaussian distribution of the expected number of struts, then the quality of OCT image is labeled as ‘poor’ because the segmentation algorithm was not able to properly detect the struts. If the percentage of images with poor quality within an OCT pullback is above a specific threshold, that pullback is classified as a ‘poor’ quality pullback. The threshold was set to 10 images (20% of the images considered for a pullback). This value was chosen because it was close to the mean number of poor quality images per pullback, which was 8.82 (17.65% of the images of a pullback).

### 2.4 Validation of the segmentation method

The results of the lumen and stent struts automatic detection algorithm were compared to manual segmentation performed by two independent expert image readers (R1 and R2). The open-source software MRIcro (University of South Carolina, Columbia, SC, USA) was used to perform the manual segmentation. While the region within the lumen contour detected in each OCT image was considered as region of interest (ROI) for lumen segmentation, the stent struts represented the ROIs for stent segmentation. For each pullback, 50 images were analyzed for both lumen and stent segmentation validation (1150 images in total). Samples were uniformly spaced inside the segments, both for lumen and stent. The validation of the automatic segmentation was performed using MATLAB 2016b.

For lumen segmentation, the following similarity indexes were computed [15]:

$$Sensitivity=\frac{TP}{TP+FN}$$ (1)

$$Specificity=\frac{TN}{TN+FP}$$ (2)

where TP is true positive, FN false negative, TN true negative and FP false positive. The calculation of these similarity indexes was done pixel-wise (i.e. each pixel was a sample). A TP was a pixel segmented in both manual and automatic segmentations. A FP was a pixel segmented only by the algorithm. A FN was a pixel segmented only by the manual reader. A TN was a pixel that was not segmented by either the manual reader or the algorithm. Furthermore, the distance between the automatic and manual segmentation was computed for each OCT image A-scan. The lumen area was also calculated for each segmented OCT image. Linear regression and Bland-Altman analysis [17] were used to evaluate the similarity between the lumen areas obtained with the different segmentation methods (automatic, R1 and R2).

For stent segmentation, sensitivity and precision (i.e. positive predicted value) were computed [9,10,15]. Sensitivity was calculated as defined in Eq 1 while precision was computed as:

$$Precision=\frac{TP}{TP+FP}$$ (3)

The calculation of these similarity indexes was done region-wise (i.e. each region was a sample). One automatically segmented region was a TP if it overlapped (at least partially) with a manually segmented region and it was a FP otherwise. A FN was a manually segmented region that did not overlap with the automatic segmentation. Additionally, the total and radial distances between corresponding stent struts were computed as defined in [15]. Briefly, the total distance is the distance between the centroid of each true positive strut of the automatic segmentation and the centroid of the closest manually segmented strut. The difference between the distances of the two centroids and the center of the lumen is the radial component of the distance.

The validation was performed by considering (i) all the OCT pullbacks and (ii) only the OCT pullbacks that were selected as ‘good’ by the OCT pullback classification algorithm. In the first case, only similarity indexes were considered as a metric to evaluate the quality of the segmentation. In the second case, all the previously described metrics were computed.

### 2.5 3D reconstruction and computational fluid dynamics

**Fig 6:**
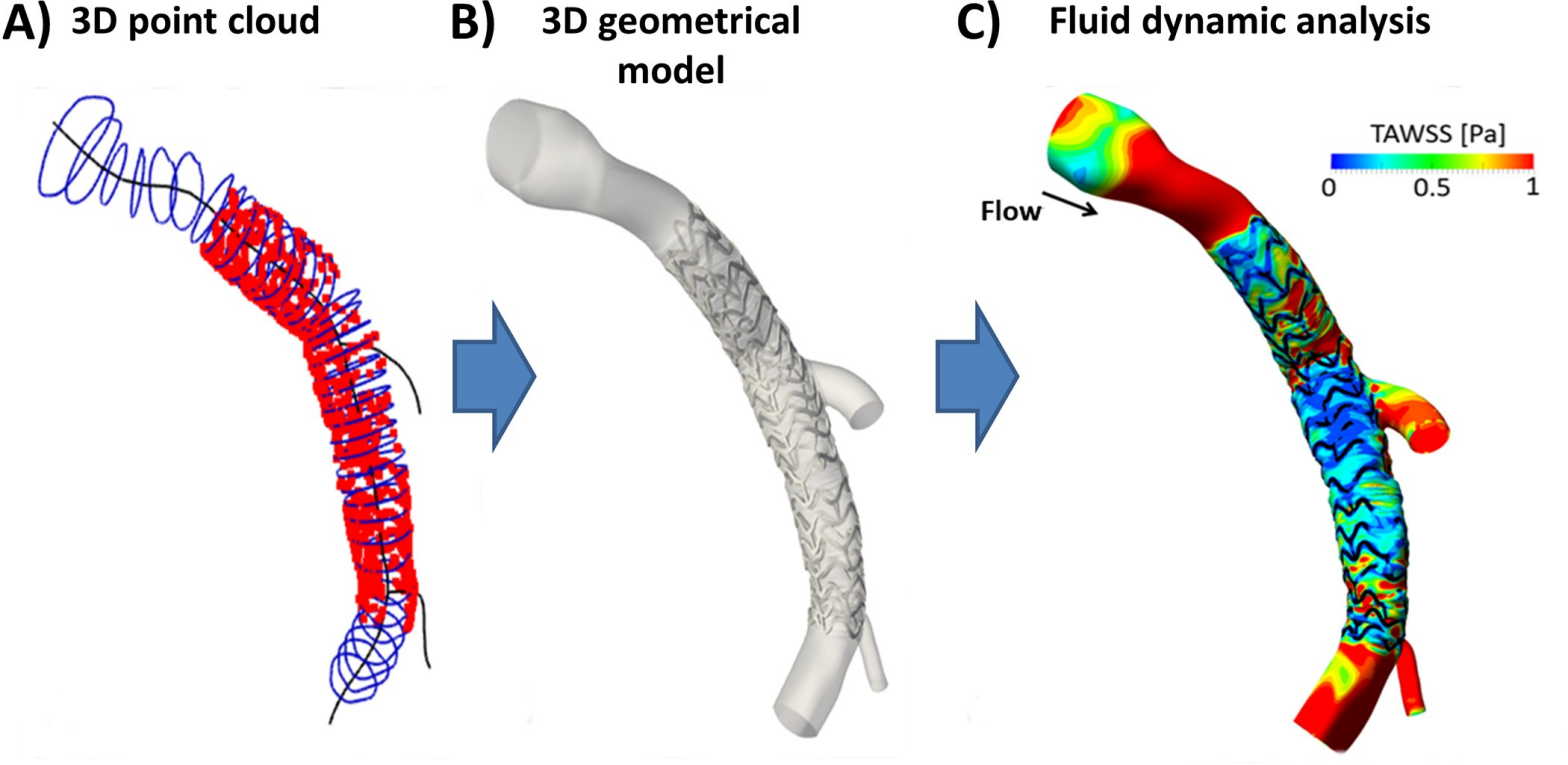
Computational fluid dynamics simulation of a left descending coronary artery treated with a 3.5x23 Absorb BVS. A) OCT lumen contours (blue) and stent struts centroids (red) positioned along the main vessel centerline (black) extracted from angiography. B) 3D geometrical model of stented coronary bifurcation. C) Contour map of time-averaged wall shear stress (TAWSS).

To demonstrate the applicability of the segmentation algorithm for the reconstruction of coronary artery geometries including a bioresorbable scaffold and its suitability for CFD applications, we performed the 3D reconstruction of one of the investigated cases. This case consists of a left anterior descending coronary artery treated with a 3.5 x 23 mm Absorb BVS. The 3D reconstruction process, which is described in detail in [18], is characterized by the following steps:

- Creation of a 3D OCT lumen contour and stent struts point cloud from 2D OCT segmented images;
- Alignment of the 3D point cloud to a reference centerline obtained by combining multiple angiographic projections (Fig 6A);
- Creation of vessel lumen surface, by interpolation of lumen contour points of the same OCT slice and subsequent loft of all lumen contour lines;
- Morphing of the stent skeleton (i.e. stent centerline) from its straight free-expanded configuration to the aligned OCT stent struts point cloud;
- Generation of the 3D patient-specific stent geometry by connecting cross-section curves placed along the deformed stent skeleton lines;
- Merging of the lumen and stent geometries to obtain the final 3D stented geometrical model (Fig 6B).

The 3D reconstructed geometry was discretized using 4,300,520 tetrahedral elements by means of ICEM CFD (ANSYS Inc., Canonsburg, PA, USA). A transient CFD analysis was performed with Fluent (ANSYS Inc.). A typical coronary artery flow waveform [19] was imposed at the inlet with a flat velocity profile. The mean inlet flow-rate (81.92 ml/min) was estimated by counting in the angiographic projections the number of frames required for the contrast agent to flow in a coronary segment [20]. A flow-split of 0.75:0.21:0.04, respectively for the distal main vessel and the two side branches, which was estimated using a diameter-based scaling law [21], was applied at the outlets. The simulation settings are reported in [22]. An example of CFD simulation output is shown in Fig 6C.

## 3. Results

This section reports the results of the validation by considering both all OCT pullbacks (Subsection 3.1) and only the OCT pullbacks that were classified as ‘good’ by the pullback classification algorithm, which were 16 out of 23 (Subsection 3.2).

### 3.1 Validation results for all OCT pullbacks

Table 1 presents the values of sensitivity and specificity for lumen contour segmentation. Table 2 displays the values of sensitivity and precision for stent strut segmentation. In both cases, the comparisons were made between: (i) the automatic segmentation and each manual segmentation and (ii) between the two manual segmentations.

**Table 1:** Performance indexes for lumen segmentation. All investigated OCT pullbacks are considered.

INDEX	AUTO vs. R1	AUTO vs. R2	R1 vs. R2
**Sensitivity**	97.42% ± 7.80%	97.31% ± 7.65%	98.53% ± 2.69%
**Specificity**	99.47% ± 0.33%	99.5% ± 0.40%	99.82% ± 0.16%

AUTO: automatic segmentation algorithm; R1: image reader 1; R2: image reader 2

**Table 2:** Performance indexes for stent segmentation. All investigated OCT pullbacks are considered.

INDEX	AUTO vs. R1	AUTO vs. R2	R1 vs. R2
**Sensitivity**	62.36% ± 26.02%	63.31% ± 26.58%	92.28% ± 14.11%
**Precision**	83.70% ± 25.52%	82.02% ± 26.28%	94.32% ± 11.85%

AUTO: automatic segmentation algorithm; R1: image reader 1; R2: image reader 2

### 3.2 Validation results for the ‘good’ quality OCT pullbacks

#### 3.2.1 Lumen detection validation

Table 3 reports the values of sensitivity and specificity for the lumen validation performed on ‘good’ quality OCT pullbacks. Both sensitivity and specificity values were similar to those found for all the 23 OCT pullbacks.

**Table 3:** Performance indexes for lumen segmentation of ‘good’ quality OCT pullbacks.

INDEX	AUTO vs. R1	AUTO vs. R2	R1 vs. R2
**Sensitivity**	98.44% ± 3.12%	98.37% ± 2.84%	98.54% ± 2.50%
**Specificity**	99.53% ± 0.34%	99.53% ± 0.38%	99.84% ± 0.15%

AUTO: automatic segmentation algorithm; R1: image reader 1; R2: image reader 2

**Fig 7:**
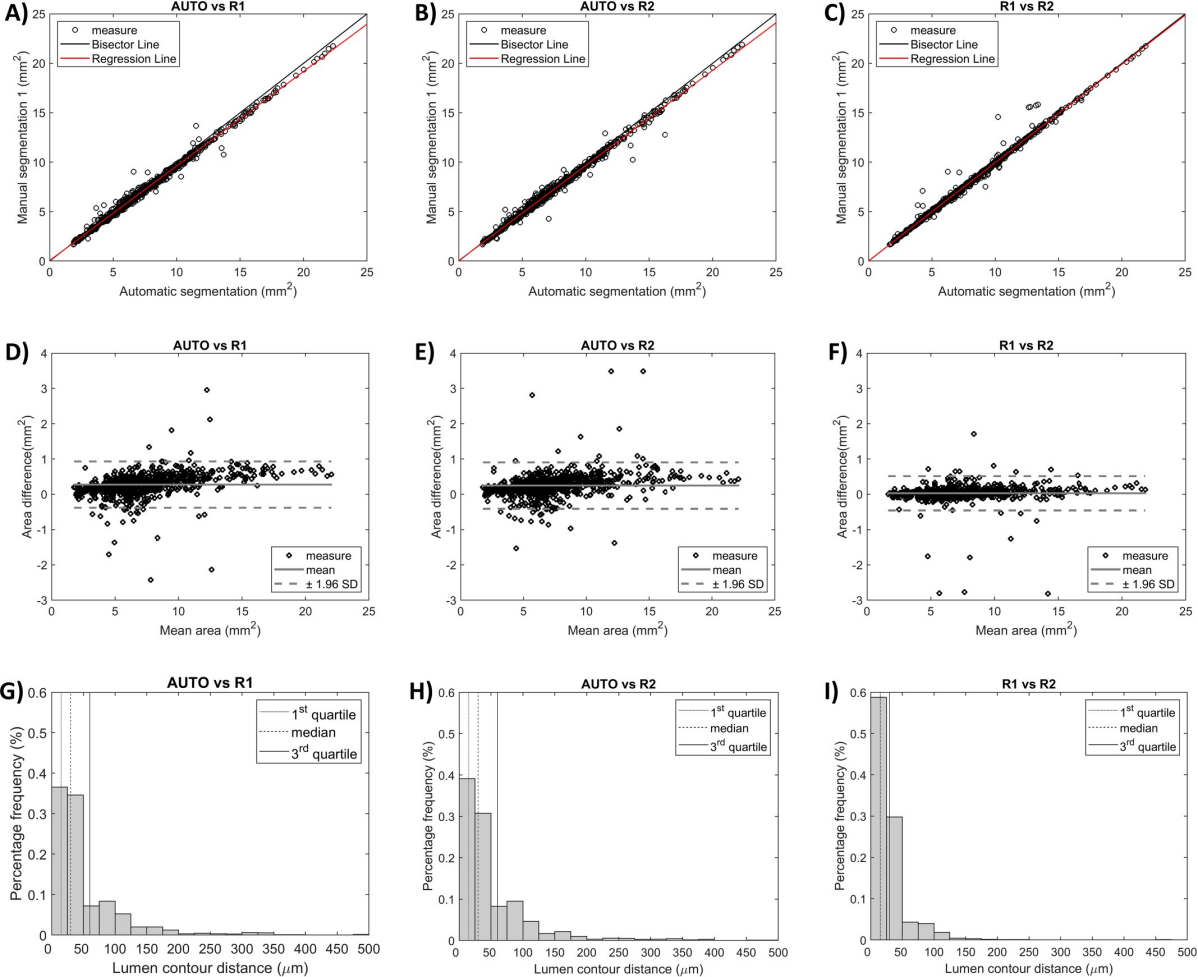
Metrics used for lumen segmentation validation. The ‘good’ quality OCT pullbacks are considered. Comparisons are made among automatic segmentation (AUTO) and manual segmentations (R1 and R2). A-C) Linear regression plots of the lumen area. D-F) Bland-Altman plots of the lumen area. G-I) Histograms showing the distribution of distances between corresponding lumen contour points (i.e. same A-scan) of different segmentations; quartiles (Q1, median, and Q3) are highlighted with different vertical lines.

The automatic segmentation showed high linear correlation with the manual one (r = 0.99, p<0.05) for both the readers (Fig 7A and 7B). The correlation coefficient *r* was comparable to that obtained for the segmentations of the two manual readers (r = 0.99, p<0.05). Fig 7D–7F displays the Bland-Altman plots comparing the automatic segmentation and the two manual segmentations. The 95% confidence interval for the error in lumen area was -0.38 mm^2^ ≤ err ≤ 0.93 mm^2^ for AUTO vs. R1, -0.41 mm^2^ ≤ err ≤ 0.91 mm^2^ for AUTO vs. R2, and -0.45 mm^2^ ≤ err ≤ 0.51 mm^2^ for R1 vs. R2. The distances between corresponding lumen contour points are compared in Fig 7G–7I. Median and quartiles (Q1 and Q3) were also computed for the distributions (Q1: 15 μm, median: 30 μm, Q3: 60 μm for AUTO vs. R1; Q1: 15 μm, median: 30 μm, Q3: 60 μm for AUTO vs. R2; Q1: 15 μm, median: 15 μm, Q3: 30 μm for R1 vs. R2).

#### 3.2.2 Stent detection validation

The values of sensitivity and precision for the lumen validation performed on ‘good’ quality OCT pullbacks is reported in Table 4. The values of both sensitivity and precision were higher than those obtained on all OCT pullbacks.

**Table 4:** Performance indexes for stent segmentation of ‘good’ quality OCT pullbacks.

INDEX	AUTO vs. R1	AUTO vs. R2	R1 vs. R2
**Sensitivity**	78.63% ± 20.13%	76.52% ± 22.51%	92.38% ± 15.03%
**Precision**	91.42% ± 15.93%	86.21% ± 20.80%	95.93% ± 9.01%

AUTO: automatic segmentation algorithm; R1: image reader 1; R2: image reader 2

**Fig 8:**
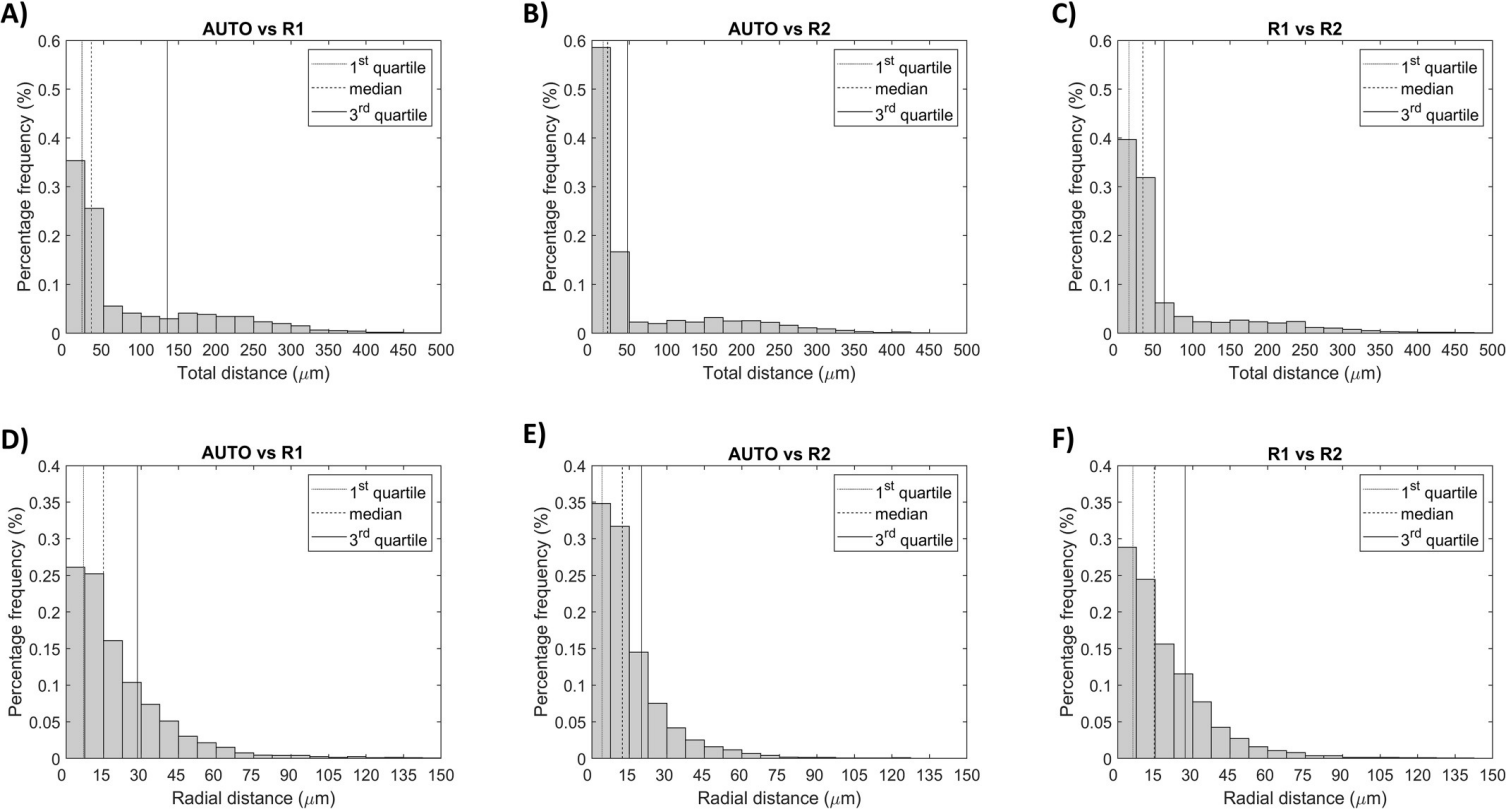
Metrics used for stent segmentation validation of the ‘good’ quality OCT pullbacks. Comparisons are made among automatic segmentation (AUTO) and manual segmentations (R1 and R2). A-C) Histograms showing the distribution of distances between corresponding stent struts. D-F) Histograms showing the radial component of the distance; quartiles (Q1, median, and Q3) are highlighted with different vertical lines.

The histograms of the distance between corresponding stent struts and their radial component are shown in Fig 8. Median and quartiles (Q1 and Q3) were also computed for the distributions. For AUTO vs R1, the median of the total distance was 33.54 μm (Q1: 21.21 μm; Q3: 135 μm), while the median of the radial distance was 14.90 μm (Q1: 7.01 μm; Q3: 28.51 μm). Similar values were observed for AUTO vs. R2, with medians of 21.21 μm and 12.22 μm for the total and radial distances, respectively (Q1: 15.00 μm and Q3: 47.43 μm for total distance; Q1: 4.11 μm and Q3: 19.88 μm for radial distance). The same can be observed for R1 vs. R2 with medians of 33.54 μm and 14.56 μm for total and radial distances, respectively (Q1: 15.00 μm and Q3: 61.85 μm for total distance; Q1: 6.07 μm and Q3: 27.02 μm for radial distance).

## 4. Discussion

In this study, a new automatic segmentation algorithm for the detection of lumen contours and polymeric bioresorbable scaffold struts in intravascular OCT images was proposed and validated. Furthermore, a method for automatic OCT pullback quality assessment was developed to classify the OCT pullbacks as ‘good’ or ‘poor’ quality cases based on the appearance of the stent struts within the OCT images. Specifically, this method classified 7 out of the 23 OCT investigated OCT pullbacks as ‘poor’ quality cases.

The validation of the automatic lumen contour segmentation algorithm performed against two expert image readers on all OCT pullbacks resulted in high values of performance indexes (sensitivity ~97% and specificity ~99%). These values were comparable to other automatic lumen detection algorithms applied to *in vivo* [23,24] or *in vitro* [15,18] OCT images with metallic stents. The 95% confidence interval for the error in lumen area computed on the ‘good’ classified OCT pullbacks was lower than that obtained in other studies analyzing *in vivo* OCT images [25,26] and similar to those analyzing *in vitro* OCT pullbacks [15,18]. Similarly to Chiastra et al. [15], the distribution of the distances between corresponding lumen contour points of the automatic and manual segmentations were skewed to the left with 75^th^ percentile equal to 60 μm, less than half the Absorb BVS strut thickness.

When considering only the ‘good’ classified OCT pullbacks, the validation of the stent strut detection algorithm resulted in a precision of ~91% and ~86% and a sensitivity of ~78% and ~76%, against the first end second expert image readers, respectively. The precision was similar to that found by Amrute et al. [9] (93%) and Cao et al. [8] (87.9%) and lower than that by Wang et al. [10] (99.2% ± 0.1%). The sensitivity was lower than that found by Amrute et al. [9] (90%), Cao et al. [8] (91.5%) and Wang et al. [10] (96.6% ± 2.0%). This last evidence can be partially explained by the fact that our detection algorithm was applied to OCT images with lower quality, in particular 8-bit images acquired with the Lunawave OCT imaging system (Terumo Corp.), as compared to the 16-bit images acquired with the C7-XR OCT system (St. Jude Medical) of the other studies [8–10]. It is also worth noting that the comparison between our two manual segmentations resulted in a sensitivity of ~92% a precision of ~96% with high standard deviation (15% and 9%, respectively). This result highlights that the quality of our OCT pullback was critical, making the detection of the stent struts difficult. Since the feature used by Amrute et al. [9] and Wang et al. [10] to define a polymeric scaffold strut is essentially based on the detection of two edges at a proper distance in the radial direction, it is reasonable to think that in OCT images characterized by noise and blood residual close to the vessel wall, such as the ones used in the present study, this feature may also be shared by other non-strut regions, leading to a drop in the specificity of the algorithm. In this study, we identified a feature that is more characteristic of a polymeric scaffold strut (i.e. bright box shape with dark core) in order to have the lowest drop in precision as possible, even in case of noise due to blood or wall protrusions. The decrease in sensitivity of the algorithm is then a trade-off for having a more precise and robust strut detection. Moreover, it is worth noting that 78% sensitivity still allowed the reconstruction of a full 3D stent geometry. Therefore, although the sensitivity was lower if compared with other studies [8–10], it was still high enough for practical applications.

The segmentation of both lumen and scaffold struts from an OCT pullback required around 5 minutes on a desktop computer equipped with CPU i7-950 @3.07 GHz and 16 GB of RAM. The processing time was comparable to the one by Amrute et al. [9], but longer than the one reported by Cao et al. [8]. However, the processing time can be dramatically reduced by converting the Matlab code to a lower level language (e.g. C++) [25] and by using graphics processing units (GPU) for the calculations [27], thus allowing for real-time applications.

Until now, the research activity on the detection of polymeric bioresorbable scaffolds in OCT images has been limited to only three works [8–10]. However, the need of automatic detection algorithms is set to increase with the continuous development and spread of the bioresorbable scaffold technology. There are multiple potential applications of our detection algorithm. More in detail, the automatic segmentation can be used to quantify lesion severity and stent malapposition [28]. In case of follow-up OCT images of percutaneous coronary intervention, the automatic lumen contour detection can be also employed to evaluate the re-endothelization after stent deployment and eventual in-stent restenosis. Additionally, as demonstrated in this study for a single case, the lumen and stent segmentation can be successfully used for the creation of patient-specific coronary artery CFD models including a high detailed geometry of the bioresorbable scaffold. The analysis of the abnormal hemodynamics caused by bioresorbable scaffolds in patient-specific anatomies is of major importance. For instance, the adverse clinical events occurred to the Absorb BVS might be partially explained by their poor hemodynamic performance, which was related to their high strut thickness [29,30].

This study is not without limitations. The detection algorithm was developed and validated on an OCT dataset of patients treated with the Absorb BVS. Although the applicability of the algorithm to other bioresorbable stent designs was not tested, the algorithm might be applied to scaffolds made of polylactic acid, such as the DESolve (Elixir Medical Corp., Milpitas, CA, USA) and the ART Pure Bioresorbable Scaffold (ART, Paris, France), which show a similar appearance in the OCT images. Moreover, as in the previous studies on bioresorbable scaffolds [8–10], our stent detection algorithm works properly only in case of ‘preserved’ box struts. Morphological closing and nonlinear filtering were used to help closing gaps in the strut boundary. Although, in general, the stent segmentation results improved, the issue remained unsolved in case a large portion of the strut boundary was missing.

## 5. Conclusions

This study presents an automatic algorithm for the detection of both lumen contours and polymeric bioresorbable scaffold struts in *in vivo* OCT images. The algorithm was validated against manual segmentation of two expert image readers using a dataset of 23 OCT pullbacks. Results of validation were good for both the lumen contour segmentation and the stent segmentation, considering the poor quality of some OCT pullbacks. Furthermore, a method for automatic OCT pullback quality assessment was proposed. Finally, one case was successfully used to show the applicability of the segmentation algorithm for the creation of patient-specific coronary artery models including the bioresorbable scaffold geometry for local hemodynamics analysis. In the future, the 3D reconstruction of many patient-specific cases treated with the Absorb BVS will enable the investigation of the role of abnormal hemodynamics on the occurrence of in-stent restenosis and thrombotic events, which limited the use of that bioresorbable scaffold.
